# High Prevalence of *Plasmodium falciparum* K13 Mutations in Rwanda Is Associated With Slow Parasite Clearance After Treatment With Artemether-Lumefantrine

**DOI:** 10.1093/infdis/jiab352

**Published:** 2021-07-03

**Authors:** Judith Straimer, Preetam Gandhi, Katalin Csermak Renner, Esther K Schmitt

**Affiliations:** 1 Novartis Institutes for Biomedical Research, Emeryville, California, USA; 2 Novartis Pharma, Basel, Switzerland

**Keywords:** artemisinin-resistance, Rwanda, R561H, *P. falciparum* malaria, artemether-lumefantrine, K13 mutations, parasite clearance half-lives, clinical study

## Abstract

In Southeast Asia, mutations in the *Plasmodium falciparum* K13 gene have led to delayed parasite clearance and treatment failures in patients with malaria receiving artemisinin combination therapies. Until recently, relevant K13 mutations had been mostly absent from Africa. Between 2018 and 2019, a phase 2 clinical study with 186 patients was conducted in Mali, Gabon, Ghana, Uganda, and Rwanda. Patients with malaria were randomized and treated with artemether-lumefantrine or cipargamin. Here we report an allele frequency of 22% for R561H in Rwanda and associated delayed parasite clearance. Notwithstanding, efficacy of artemether-lumefantrine remained high in Rwanda, with a 94.4% polymerase chain reaction–corrected cure rate.

## Teaser

A phase 2 clinical study was conducted in Mali, Gabon, Ghana, Uganda, and Rwanda. Patients with malaria were randomized to artemether-lumefantrine or cipargamin. Here we report an allele frequency of 22% for R561H in Rwanda and associated delayed parasite clearance.

Artemisinin and its derivatives remain the cornerstone of antimalarial combination therapies, but evolution of drug resistance could limit treatment options and challenge global malaria elimination efforts. Artemisinin resistance emerged in Southeast Asia and has since spread across the subcontinent [1]. It is mediated by mutations in the propeller domain of the *Plasmodium falciparum* K13 (Kelch 13) protein and manifests in patients with malaria as a parasite clearance half-life (PCT_½_) >5 hours [2, 3].

The K13 protein is highly polymorphic, and a previous study described the distribution of 108 different K13 alleles worldwide [4]. Most notable was that artemisinin resistance-conferring mutations are highly prevalent in Southeast Asia but absent from other malaria-endemic regions. However, at least 2 more recent studies reported the K13 mutation R561H in patients from Rwanda and Tanzania [5, 6]. In Southeast Asia, this allele is associated with delayed parasite clearance in patients and increased parasite survival in vitro [3, 5]. Here we confirm the previously reported emergence of R561H allele in Rwanda and associate mutant K13 alleles, including 2 novel mutations, with slow parasite clearance in patients.

## METHODS

This was a multicenter, randomized, open-label, dose escalation phase II trial, conducted in Mali, Gabon, Ghana, Uganda, and Rwanda, with support from Novartis and the Wellcome Trust. Eligible patients were adults (≥18 years old and ≥45 kg body weight) with microscopic confirmation of acute uncomplicated *Plasmodium falciparum (P. falciparum)* malaria (parasitemia of 500–50 000/μL with axillary temperature ≥37.5ºC, oral, tympanic, or rectal temperature ≥38.0ºC, or history of fever during the previous 24 hours).

Patients were randomized and treated in 5 cohorts, using ascending single or multiple doses of cipargamin. Artemether-lumefantrine (AL; 80/480 mg; twice daily for 3 days) was used as an active comparator in each cohort. Patients receiving cipargamin who met protocol-specified treatment failure criteria received AL as rescue medication. Blood samples were taken for parasite counts (Giemsa-stained thick and thin films) at baseline, then at 2, 4, 8, 12, 24, 36, 48, 60, and 72 hours and days 4, 7, 10, 14, 21, and 28 after starting treatment, and at unscheduled visits.

At least 200 thick film fields were examined. Parasite counts were made per 200 leukocytes (or if the count was <100 parasites, counting was continued for up to 500 leukocytes). Slides were read by 2 trained microscopists, and average values were used to calculate parasitemia levels. Samples for polymerase chain reaction (PCR) genotyping of *P. falciparum*, to assess recrudescence versus reinfection and identify specific resistance markers in PfATP4 (PF3D7_1211900) and K13 (PF3D7_1343700) genes, were taken at baseline, at days 7, 10, 14, 21, and 28, and at the time of treatment failure. Slopes for PCT_½_ were calculated for each patient using the R programs developed by the World Wide Antimalarial Resistance Network 2015, with modification to lower the initial parasite count from 1000/µL to 500/µL, as the 500/µL is the minimal inclusion criterion in this study. Trial procedures relating to informed consent, dose escalation, and trial oversight are detailed elsewhere (ClinialTrials.gov NCT03334747; reported 25 September 2020).

## RESULTS

The study CKAE609A2202 was conducted in 5 African countries with high malaria transmission (Mali, Gabon, Ghana, Uganda, and Rwanda), using uniform and quality-controlled procedures for parasite counting. Between February 2018 and October 2019, 186 patients with uncomplicated *P. falciparum* malaria were randomized and treated with either AL (n = 51) or cipargamin (n =135). Efficacy results and results of safety and resistant marker analysis for cipargamin are reported elsewhere. The sequence of the propeller domain of the *k13* gene was PCR amplified from 184 samples at baseline and analyzed for K13 mutations. According to recommendations from the World Health Organization, the sequences were searched for validated, candidate, and novel K13 mutations [7]. Validated mutations have demonstrated clinical evidence of delayed parasite clearance in patients, and reverse genetic experiments have established increased parasite survival after artemisinin exposure in vitro. Candidate mutations lack genetic validation but are associated with delayed parasite clearance in clinical trials [7].

**Figure 1.**
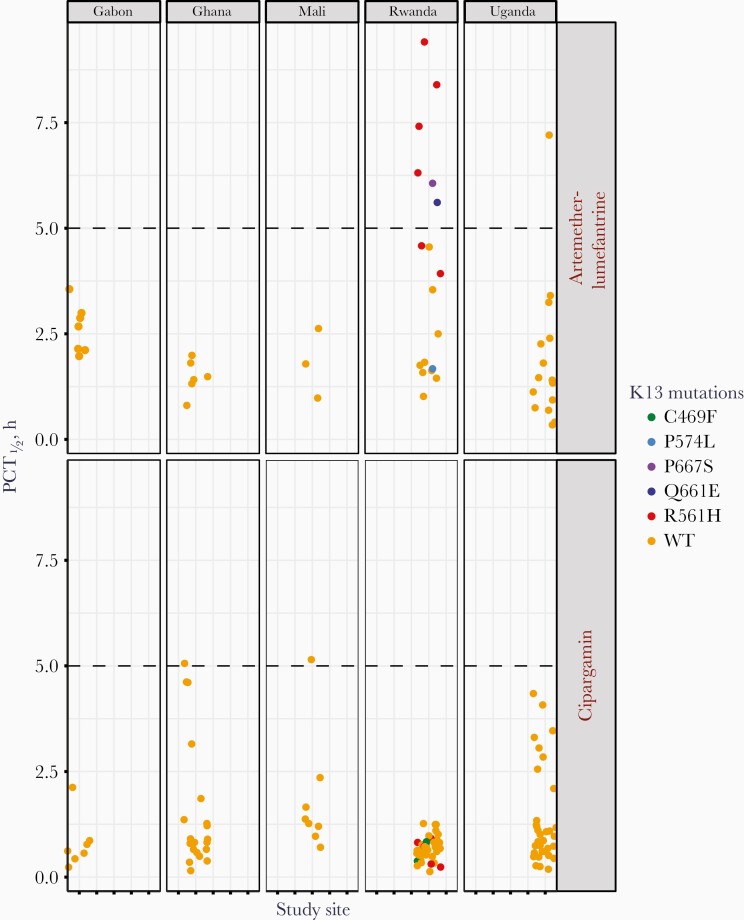
K13 mutations are associated with longer parasite clearance half-life (PCT_½_) after treatment with artemether-lumefantrine. K13 alleles are plotted against the observed PCT_½_ in patients. The figure shows patients treated with artemether-lumefantrine (*upper panel*) or cipargamin (*lower panel*). Mutant alleles were exclusively found in Rwanda and were associated with slower parasite clearance in patients treated with artemether-lumefantrine but not in those treated with cipargamin. Cipargamin was given in single doses of 10–150 mg or multiple doses (3 days) of 10–50 mg, which accounts for the observed range of PCT_½_. Abbreviation: WT, wild type.

In Mali, Gabon, Ghana, and Uganda, all 111 patients were infected with K13 wild-type parasites (Figure 1). At 2 study sites in Kigali City, Rwanda, 22 mutant K13 alleles were identified in 73 patients (30%), and the validated mutation R561H appeared in 16 cases (21.9%). The validated mutation P574L was present in 1 patient and the candidate mutation C469F in 3 patients (Figure 1). In 2 patients, we discovered a novel mutation, Q661E and P667S (Table 1). The presence of R561H mutation in Rwanda has been reported elsewhere, but a higher allele frequency was observed in this study. Previous publications describe a clinical study conducted in Rwanda between 2012 and 2015 that included treatment with AL and genotyping of the K13 gene [5, 8]. The authors reported a prevalence of 7.4% for R561H in Masaka, a city 20 km southeast of Kigali.

**Table 1.** Proportions of Artemether-Lumefantrine–Treated Patients With a Parasite Clearance Half-life >5 Hours or Parasitemia on Posttreatment Day 3, by K13 Genotype

Population Studied	Patients, No. (%)			
	PCT_½_ >5 h	>100 Parasites /µL on d 3	<100 Parasites/µL on d 3	No Parasites on d 3
All AL (n = 51)	7 (14)	1 (2)	4 (8)	46 (90)
K13 WT (n= 41)	1 (2)	0 (0)	0 (0)	41 (100)
K13 R561H (n = 6)	4 (67)	1 (17)	3 (50)	2 (33)
K13 P574L (n = 1)	0 (0)	0 (0)	0 (0)	1 (100)
K13 Q661E (n = 1)	1 (100)	0 (0)	0 (0)	1 (100)
K13 P667S (n = 1)	1 (100)	0 (0)	1 (100)	0 (0)

Abbreviations: AL, artemether-lumefantrine; PCT_½_, parasite clearance half-life; WT, wild-type.

In 2019, a second surveillance report from Huye province, 100 km south of Kigali, identified R561H in 3 of 66 patients (4.5%) [9]. A World Health Organization report documented an allele frequency of 11.9% for R561H in Rwanda [7]; that analysis was based on 219 samples collected in 2018. Here, we report an allele frequency of 22% for R561H in 73 samples collected between November 2018 and October 2019 in Kigali City. The absence of K13 mutant parasites from study sites in other countries and their relatively high prevalence in Rwanda suggest a unique and recent selection pressure. Since 2006, AL has been used in Rwanda to control malaria [10].

To determine whether the presence of K13 mutations affected the speed of parasite reduction, parasite clearance was measured by 2 parameters: PCT_½_ and positive parasitemia 3 days after treatment (Figure 1 and Table 1). In our study, 51 patients received AL across 5 countries, but only 9 of these patients were infected with parasites carrying mutations in the K13 gene. Despite the small numbers, we observed a correlation between K13 alleles and parasite clearance.

In Mali, Gabon, Ghana, and Uganda, all parasite infections except 1 were fast clearing, and this finding is consistent with the presence of wild-type K13 alleles in these countries (Supplementary Table 1). In Rwanda, 6 of 18 patients (33%) met the criteria for clinical artemisinin resistance, with PCT_½_ >5 hours (Figure 1). Four were infected with R561H parasites, and 2 with parasites that carried either of the novel mutations Q661E or P667S. Parasite clearance times >5 hours were correlated with a positive parasite smear on day 3 (Supplementary Table 2). In the patient infected with Q661E, the parasitemia had cleared by day 3 after treatment, but in the other patients with delayed parasite clearance a positive smear was still recorded on day 3. In the remaining 3 patients infected with mutant K13 parasites, the parasitemia cleared quickly (PCT_½_ <5 hours) and the infections did not meet the definition of clinical artemisinin resistance. Other infections with mutant K13 alleles were treated with cipargamin, and parasite clearance times were not affected (Figure 1).

This confirms the previously reported potency of cipargamin against parasites with K13 mutations in Asia [11]. Despite the observed delay in parasite clearance in some patients, the efficacy for AL in this study remained high, with day 28 PCR-adjusted adequate clinical and parasitological responses of 94.4% in Rwanda and 100% in Mali, Gabon, Ghana, and Uganda (Supplementary Table 3).

## Discussion

Our findings of delayed parasite clearance contrast with results published by Uwimana et al in 2019 [8]; they measured parasitemia on day 3 after treatment and, despite the presence of validated K13 mutations in Rwanda, were unable to identify slow-clearing infections between 2012 and 2015. Here, we show evidence of slow parasite clearance in Africa associated with mutant K13 alleles in 6 of 9 patients based on PCT_½_ >5 hours and 5 of 9 based on parasitemia on day 3. While the clinical relevance of R561H is established, the 2 newly identified mutations Q661E and P667S require further investigation including *Plasmodium* gene editing to confirm the impact of these mutations on ring-stage survival and fitness in vitro [3, 12]. However, it is striking that both were associated with PCT_½_ >5 hours. The observed deviations from expected PCT_½_ for 1 parasite carrying the WT allele (Uganda) and 3 carrying validated mutations in the K13 gene are in line with previously reported data [3].

Patient-to-patient variability in PCT_½_ could be attributed to differences in drug exposure and underlying immunity acquired through repeated malaria infections [13]. The impact of immunity on parasite clearance could be significant, considering the adult patient population included in this study. The overall efficacy of 98% in the study is consistent with other AL efficacy studies in Africa, including the findings published by Uwimana et al [8], who found a day 28 PCR-adjusted adequate clinical and parasitological response of 98.3%. In contrast to some of the above-mentioned studies, ours is a multicenter study involving multiple countries and unified procedures across clinical sites, so the differences in prevalence of the K13 mutations and the associated delayed parasite clearance in Rwanda compared with the other African countries are significant.

A clear limitation of the current study is its small size, especially in the geographic subgroup of Rwanda, which comprised 73 patients (18 receiving AL). Nevertheless, the high prevalence of K13 mutant allele frequency and slow parasite clearance warrant further investigations in controlled clinical trials. In patients infected with parasites carrying mutations in the K13 gene, the reduced efficacy of artemisinin increases the pressure on the partner drug to clear the infection. This has been detrimental to several combinations in Southeast Asia, where Artemisinin Combination Therapies (ACTs) are failing at alarming rates [14]. In Cambodia, Thailand, and Vietnam the overall PCR-corrected efficacy for dihydroartemisinin-piperaquine was reported to be 48% [14]. In contrast, ACTs remain efficacious in Africa, and the prevalence of K13 mutations in Southeast Asia has not compromised the efficacy of AL [7]. The sustained high efficacy of AL in particular can be attributed to an absence of known resistance to lumefantrine [15]. However, our findings highlight the need for consistent monitoring of K13 resistance markers in Africa to ensure the efficacy of ACTs on the continent, while new therapies with novel mode of actions are in development.

## Supplementary Data

Supplementary materials are available at The Journal of Infectious Diseases online. Consisting of data provided by the authors to benefit the reader, the posted materials are not copyedited and are the sole responsibility of the authors, so questions or comments should be addressed to the corresponding author.

jiab352_suppl_Supplementary_Data

## Notes

***Acknowledgments.*** We thank all patients volunteering to participate in the study and the staff and investigators at the clinical sites. Christian Nsanzabana and his team from SwissTPH performed the genotyping and resistance marker sequence analysis of the clinical samples. Finally, we thank Caroline Boulton and Thierry Diagana for providing valuable comments on the first draft of this manuscript and all colleagues from the malaria global program team for their support.

***Author contributions.*** J. S. and E. K. S. conceived the study, analyzed the data, and wrote the first draft of the manuscript. P. G. and K. C. R. edited the manuscript.

***Financial support.*** This work was supported by the Wellcome Trust (grant 207813/Z/17/Z) and by Novartis.

***Potential conflicts of interest.*** All authors are employed by Novartis and shareholders of Novartis Pharma. All authors have submitted the ICMJE Form for Disclosure of Potential Conflicts of Interest. Conflicts that the editors consider relevant to the content of the manuscript have been disclosed.
