# Supplementary material for: High Prevalence of Plasmodium falciparum K13 Mutations in Rwanda Is Associated With Slow Parasite Clearance After Treatment With Artemether-Lumefantrine
Source: J Infect Dis. 2021 Jul 3;225(8):1411–4. doi: 10.1093/infdis/jiab352 (PMC9016418; doi:10.1093/infdis/jiab352)
Supplement: jiab352_suppl_Supplementary_Data [file jiab352_suppl_supplementary_data.docx]

**Supplementary Data**

**High prevalence of *P. falciparum* K13 mutations in Rwanda is associated with slow parasite clearance after treatment with artemether-lumefantrine**

Authors: Judith Straimer, Preetam Gandhi, Katalin Csermak Renner, Esther K Schmitt*

**Online methods**

Trial design and oversight

This was a multi-center, randomized, open-label, dose escalation Phase II trial, conducted in Mali, Gabon, Ghana, Uganda, and Rwanda with support from Novartis and the Wellcome Trust. The Independent Ethics Committee or Institutional Review Board reviewed the trial protocol and all amendments for each center. The trial was conducted according to ICH E6 Guidelines for Good Clinical Practice that have their origin in the Declaration of Helsinki.

Patients

Eligible patients were adults (≥ 18 years old and ≥ 45 kg body weight) with microscopic confirmation of acute uncomplicated *P. falciparum* malaria (parasitemia of 500 to 50,000/μL with axillary temperature ≥ 37.5ºC or oral/tympanic/rectal temperature ≥ 38.0ºC or history of fever during the previous 24 hours). Exclusion criteria included mixed *Plasmodium* infections, and severe malaria according to World Health Organization (WHO) criteria among others.

Trial procedures

Trial procedures relating to the dose escalation process and decision criteria are detailed elsewhere [1,2]. Patients were treated in five cohorts, using ascending single or multiple doses of cipargamin. Patients were randomized to treatment groups, cohort by cohort, with a pause between cohorts for safety review. Interactive Response Technology (IRT) was used for randomization. Artemether-lumefantrine (80/480 mg, b.i.d for 3 days) was used as an active comparator in each cohort.

Patients received close monitoring in an inpatient setting for at least the first three days, followed by frequent outpatient monitoring for a total of four weeks. Patients were required to yield two consecutive trial assessments with negative blood smear for *P. falciparum* parasites and clearance of fever in order to be discharged. Cipargamin patients who met protocol-specified treatment failure criteria received artemether-lumefantrine as rescue medication.

Blood samples were taken for parasite counts (Giemsa-stained thick and thin films) at baseline, then at 2, 4, 8, 12, 24, 36, 48, 60, and 72 hours, then days 4, 7, 10, 14, 21 and 28 after starting treatment, and at unscheduled visits. At least 200 thick film fields were examined. Parasite counts were made per 200 leukocytes (or if the count was <100 parasites, counting was continued for up to 500 leukocytes). Microscopists received training and passed proficiency testing according to WHO guidelines. Slides were read by two microscopists and average values were used to calculate parasitemia levels. Samples for PCR genotyping of *P. falciparum*, to assess recrudescence versus reinfection and identify specific resistance markers in PfATP4 and K13 genes, were taken at baseline and days 7, 10, 14, 21, and 28, and at the time of treatment failure.

Parasite clearance half-live

Parasite clearance slope half-life for parasite clearance were calculated for each subject using the WWARN (World Wide Antimalarial Resistance Network 2015) Parasite Clearance Estimator [3]. Parasite clearance slope half-life for parasite clearance were calculated using the R programs developed by WWARN with modification to lower the initial parasitemia from 1000 parasites/µl to 500 parasites/µl as the 500 parasites/µl is the minimal inclusion criterion in this study. It's noted that parasite clearance rate constant and slope half-life for parasite clearance cannot be calculated if a patient has less than 3 non-zero parasite counts or the parasite count decreases quickly from >=1000 to below the limit detection.

**References**

1. Schmitt EK, Ndayisaba G, Yeka A et al. Efficacy of cipargamin (KAE609) in a randomized, Phase II dose-escalation study in adults in sub-Saharan Africa with uncomplicated *Plasmodium falciparum* malaria. 2021; Submitted.

2. Ndayisaba G, Yeka A, Asante KP, et al. Hepatic safety and tolerability of cipargamin (KAE609), in adult patients with *Plasmodium falciparum* malaria: a randomized, phase II, controlled, dose-escalation trial in sub-Saharan Africa. 2021; Submitted.

3. Flegg JA, Guerin PJ, White NJ, Stepniewska K. Standardizing the measurement of parasite clearance in *falciparum* malaria: The parasite clearance estimator. Malar J. 2011; 10:339.

**Supplementary Tables**

###### **Supplementary Table 1**. Proportion of patients with K13 mutations at baseline by geographic location across all treatment arms (N=184)

| K13 genotype | All patients (N=184)  n (%) | Rwanda  (N=73)  n (%) | Mali, Gabon, Ghana, Uganda  (N=111)  n (%) |
| --- | --- | --- | --- |
| R561H | 16 (8.7) | 16 (21.9) | 0 (0) |
| P574L | 1 (0.5) | 1 (1.4) | 0 (0) |
| C469F | 3 (1.6) | 3 (4.1) | 0 (0) |
| Q661E | 1 (0.5) | 1 (1.4) | 0 (0) |
| P667S | 1 ( 0.5) | 1 (1.4) | 0 (0) |
| WT | 162 (88) | 51 (69.9) | 111 (100) |

**Supplemental Table 2.** Proportion of AL patients with delayed clearance by geography

| Event of delayed parasite clearance | All patients (N=51)  n (%) | Rwanda (N=18)  n (%) | Mali, Gabon, Ghana, Uganda (N=33) n (%) |
| --- | --- | --- | --- |
| PCT_1/2_ >5h | 7 (13.7) | 6 (33.3) | 1 (3) |
| Parasitemia Day 3 | 5 (9.8) | 5 (27.8) | 0 (0) |
| Parasitemia Day 3 >100 p/µl | 1 (2) | 1 (5.6) | 0 (0) |
| Parasitemia Day 3 <100 p/µl | 4 (8) | 4 (22.2) | 0 (0) |

**Supplementary Table 3.** Proportion of AL patients with treatment failure by day 28

| Treatment failure | All patients  (N=51) n (%) | Rwanda (N=18)  n (%) | Mali, Gabon, Ghana, Uganda  (N=33) n (%) |
| --- | --- | --- | --- |
| Overall efficacy uncorrected | 49 (96.1) | 16 (88.9) | 33 (100) |
| Overall efficacy corrected* | 50 (98) | 17 (94.4) | 33 (100) |
| All late TF | 2 (4) | 2 (11.2) | 0 (0) |
| Recrudescence | 1 (2) | 1 (5.6) | 0 (0) |
| New infection | 1 (2) | 1 (5.6) | 0 (0) |

*PCR-corrected based on genotyping of parasites at baseline and day of recurrence
